# Development of a loop-mediated isothermal amplification (LAMP) assay for rapid screening of ticks and fleas for spotted fever group rickettsia

**DOI:** 10.1371/journal.pone.0192331

**Published:** 2018-02-01

**Authors:** Bruce H. Noden, Jaclyn Martin, Yisel Carrillo, Justin L. Talley, Francisco M. Ochoa-Corona

**Affiliations:** Department of Entomology and Plant Pathology, Oklahoma State University, Stillwater, Oklahoma, United States of America; University of Minnesota, UNITED STATES

## Abstract

**Background:**

The importance of tick and flea-borne rickettsia infections is increasingly recognized worldwide. While increased focus has shifted in recent years to the development of point-of-care diagnostics for various vector-borne diseases in humans and animals, little research effort has been devoted to their integration into vector surveillance and control programs, particularly in resource-challenged countries. One technology which may be helpful for large scale vector surveillance initiatives is loop-mediated isothermal amplification (LAMP). The aim of this study was to develop a LAMP assay to detect spotted fever group (SFG) rickettsia DNA from field-collected ticks and fleas and compare with published end-point PCR results.

**Methodology/Principal findings:**

A Spotted Fever Group rickettsia-specific loop-mediated isothermal amplification (SFGR-LAMP) assay was developed using primers based on a region of the *R*. *rickettsii* 17kDa protein gene. The sensitivity, specificity, and reproducibility of the assay were evaluated. The assay was then compared with the results of end-point PCR assays for pooled tick and flea samples obtained from field-based surveillance studies. The sensitivity of the SFGR-LAMP assay was 0.00001 ng/μl (25μl volume) which was 10 times more sensitive than the 17kDa protein gene end-point PCR used as the reference method. The assay only recognized gDNA from SFG and transitional group (TRG) rickettsia species tested but did not detect gDNA from typhus group (TG) rickettsia species or closely or distantly related bacterial species. The SFGR-LAMP assay detected the same positives from a set of pooled tick and flea samples detected by end-point PCR in addition to two pooled flea samples not detected by end-point PCR.

**Conclusions/significance:**

To our knowledge, this is the first study to develop a functional LAMP assay to initially screen for SFG and TRG rickettsia pathogens in field-collected ticks and fleas. With a high sensitivity and specificity, the results indicate the potential use as a field-based surveillance tool for tick and flea-borne rickettsial pathogens in resource-challenged countries.

## Introduction

The importance of tick and flea-borne rickettsia infections is increasingly recognized worldwide as a principal cause of non-malarial febrile illness [1–4]. There are more than 25 bacteria, including species in the genera *Anaplasma*, *Ehrlichia*, *Orientia*, and *Rickettsia*, impacting human and animal health that are transmitted by vector-borne arthropods, mainly ticks or fleas [2]. While most are treatable with antibiotics, several are difficult to diagnose at low infection levels [5,6]. Reports continue to demonstrate that these tick- and flea-borne pathogens are impacting human and animal populations in low resource countries which lack technical and material capabilities. Often, these countries are not aware that these pathogens are circulating in their populations [1,3,7,8]. In recent years, increased funding and research focus has shifted to the development of point-of-care diagnostics that will detect the causative agents of neglected tropical diseases [6]. While some of the diagnostics show promise in the detection of vector-borne bacterial pathogens in their arthropod vectors [5], little research effort has been devoted to their integration into vector surveillance and control programs, particularly in resource-challenged countries.

There are challenges to address in order to integrate some of these novel detection assays into vector-borne disease surveillance programs. After the collection of useful samples, the testing of field-collected ticks and fleas normally involves time-consuming and expensive DNA extraction protocols followed by testing the samples using molecular assays, most of which depend on expensive equipment, trained personnel, and access to funding to cover the expensive extraction kits, molecular probes, and post-screening assays required to determine the species detected [9]. One technology which may be useful for large scale vector surveillance, possibly by alleviating some of these expenses, is loop-mediated isothermal amplification (LAMP).

Originally developed in 2000 [10], the basis of this assay uses a DNA polymerase, which operates in isothermal conditions, along with 4–6 primers which form a loop structure which precipitates in the reaction mixture. LAMP has been described as rapid, robust, simple, sensitive, and highly specific [11]. Important for working with vector-borne pathogens in arthropods, it can amplify DNA in partially processed samples [12] as compared with end-point polymerase chain reaction (PCR) and quantitative PCR (qPCR). LAMP is reported to be less likely altered by interfering inhibitors commonly found in ticks [13]. While many studies have recorded the development of LAMP assays to detect rickettsial pathogens in humans and animals, only two studies in the literature have assessed the prevalence of bacterial pathogens in field-collected ticks as part of a surveillance program [13,14]. The aim of this study is to develop a LAMP assay to detect SFG rickettsia DNA from field-collected ticks and fleas and compare the results with those from established end-point PCR assays.

## Methods

### DNA preparation

DNA samples, including positive controls, used in this study were cell culture stocks or genomic DNA from 14 bacteria species generously provided by different laboratories: *Rickettsia rickettsii*, *Ehrlichia chaffeensis*, and *Anaplasma phagocytophilum* (Dr. William Nicholson (CDC Atlanta)); *A*. *phagocytophilum* (Dr. Katherine Kocan and Dr. Edmour Blouin (Oklahoma State University)); *R*. *amblyommatis* (previously *R*. *amblyommii* [15], *Rickettsia montanensis*, *Rickettsia parkeri*, and *Coxiella burnetii* (Dr. Ed Shaw (Oklahoma State University)); *Rickettsia conorii* and *Rickettsia africae* (Dr. Ju Jiang and Dr. Allen Richards (Rickettsial Diseases Research Program, Naval Medical Research Center, Silver Spring, MD)); *Rickettsia typhi* (a Typhus Group (TG) rickettsia [16]) and *Rickettsia felis* (a Transitional group (TRG) rickettsia [16]) (Dr. Abdu Azad and Magda Beier-Sexton (University of Maryland Baltimore)); strains of *Salmonella typhi* and *Escherichia coli* (Dr. Li Ma (Oklahoma State University)). All work with bacterial DNA was covered under the Institutional Biosafety Committee (IBC protocol 15–1) at Oklahoma State University.

### End point polymerase chain reaction amplification

End-point PCR was performed using previously described SFG rickettsia primers which amplify a portion of the 17kDa protein gene [17,18]. The 17kDa protein gene was chosen as it is an important surface protein gene unique to *Rickettsia* spp and used in the development of other molecular screening assays for PCR and qPCR [19,20]. The results from the testing of pools of field-collected ticks and fleas by end-point PCR assays were previously reported [21,22]. A portion of the pools from these published studies were used for comparison and screening of the same pooled samples of field-collected ticks and fleas using this SFGR-LAMP assay. For end-point PCR assays, positive controls consisted of gDNA of *R*. *rickettsia* and negative controls were non-template controls (NTC). Controls were used in each PCR assay and for optimization purposes, positive and negative controls were replicated three times with the main assays.

### LAMP methods

**Fig 1:**
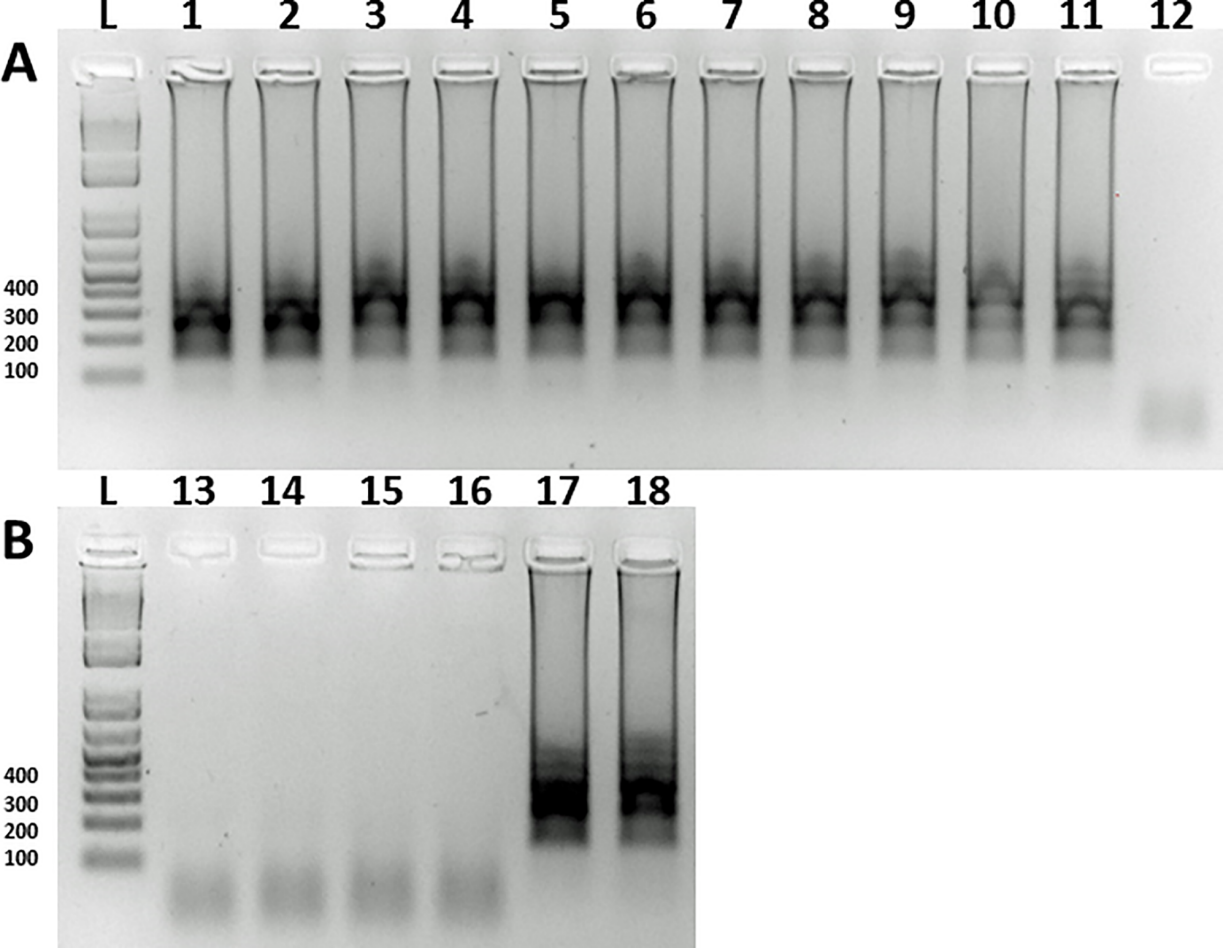
**Gel electrophoresis of SFGR-LAMP amplification products after using GspSSD2.0 Isothermal Master Mix (A & B)**. Sensitivity of LAMP assay for detection of SFG rickettsia DNA using ten-fold serial dilutions of a recombinant plasmid containing the target sequence of *Rickettsia rickettsii* 17kDa protein gene. Each dilution was duplicated starting at 1ng (lane 1 & 2), 0.1ng (lane 3 & 4), 0.01ng (lane 5 & 6), 0.001ng (1pg) (lane 7 & 8), 0.0001ng (lane 9 & 10), 0.00001ng (lane 11 & 12), 0.000001ng (1fg) (lane 13 & 14). Lane L is a 1Kb ladder. Lanes 15–18 are duplicate tests of two randomly chosen tick pools (Pool 17 (lane 15 and 16) and Pool 33 (lane 17 and 18).

Six primers were designed for LAMP assay (Table 1) and were designed based on the 17kDa protein gene, the same region that has been used for end-point and qPCR primers to screen SFG rickettsia species in field-collected ticks [20,23]. Primer Explorer version 4 (Fujitsu Ltd, Japan) was used to design the LAMP primers (Fig 1) and the alignment of the 17kDa protein gene targeted conserved sequences was performed using ClustalW2 (EMBL-EBI, UK). The following 17kDa protein gene sequences retrieved from GenBank (*R*. *rickettsii*, *R*. *amblyommatis*, *R*. *parkeri*, *R*. *montanensis*, and ‘*Candidatus* R. andeanae’) were used for consensus analysis and primer design: KC464548, KC107823, GU723477, GU723476, DQ176856, AY189818, J03371, AY281069, KC713872, U11013, DQ517291, EU828788, EF689730, JN378400, EF102237, U17008, EF689732, GU395295, and DQ402377. Three of the LAMP primers (FIP, BIP and Loop B) overlap regions of the 17kDa protein gene qPCR primer set and TaqMan probe described by Jiang et al. [20]. Two commercially available LAMP chemistries were used: the *Bst* 2.0 WarmStart DNA Polymerase (New England Biolabs, USA) and the GspSSD2.0 Isothermal Master Mix (ISO-004) (Optigene, UK). For *Bst* 2.0 polymerase, the MgCO_4_ and Betaine concentrations and the optimal isothermal temperature were achieved after rounds of preliminary optimization. The GspSSD2.0 Master Mix from Optigene did not required optimization since it was used according to the manufacturer’s instructions. Amplicons from F3/B3 LAMP primers were purified using a PCR purification kit (Qiagen) and sequenced in the Oklahoma State University core facility to confirm that the targeted region amplified by the LAMP assay matched with the expected 17kDa protein gene of *R*. *rickettsii*.

**Table 1:** SFGR-LAMP primer sequences targeting the spotted fever group *Rickettsia* sp. 17kDa protein gene.

Name of primer	Primer Sequence (5’-3’)
Rr17F3	5’-TGT TAC AAG CCT GTA ACG G -3’
Rr17B3	5’- TCC TGT TCA TCC ATA CCT G -3’
Rr17FIP	5’- GAG AAC CAA GTA ATG CGC CGG GCG GTA TGA ATA AAC AAG G -3’
Rr17BIP	5’- AAT TCG GTA AGG GCA AAG GAC CAC CGA TTT GTC CAC CAA -3’
Rr17LoopF	5’- CCG CCA AGA AGT GTT CCT GTA -3’
Rr17LoopB	5’-AGC TTG TTG GAG TAG GTG TAG GTG -3’

### *Bst* 2.0 LAMP

Each LAMP reaction totaled 25 μl consisting of 9 μl nuclease free water, 2.5 μl 10x Isothermal Amplification Buffer Pack (contains 20mM Tris-HCl, 10mM (NH_4_)_2_SO_4_, 50mM KCl, 2mM MgSO_4_, 0.1% Tween^®^ 20) (New England Biolabs), 3.5 μl 10 mM each dNTPs (New England Biolabs), 1 μl 100 mM MgSO_4_ (New England Biolabs), 3.5 μl 5M Betaine (Affymetrix), and 2.5 μl 10x primer mix (2μM F3 primer, 2μM B3 primer, 16μM FIP, 16μM BIP, 8μM LoopF and 8μM LoopB), 1.0 μl *Bst* 2.0 WarmStart DNA Polymerase (8 units/μl) (New England Biolabs), 1.0 μl 3mM Hydroxynaphthol blue (HNB) (Sigma-Aldrich), and 1 μl of sample DNA. Mineral oil was added on top of each reaction tube (25 μl) to minimize contamination for a total of 50 μl per reaction. LAMP amplification reactions were at 65° C for one hour in a GeneMate Mini Dry Bath with heated lid (BioExpress) followed by five minutes at 95° C to stop the reaction. A positive control (*R*. *rickettsii* gDNA) and a non-template control (NTC, water) were included in each LAMP assay. After incubation, the tubes containing the amplified DNA target were scored visually by two persons, one of which was completely blinded to the assay setup for control purposes; dark blue to purple corresponded to a negative reaction (absence of amplification) and light blue positive. To confirm the reaction, 25μl of each LAMP product was loaded into a 2% agarose gel stained with ethidium bromide and visualized after electrophoresis.

### GspSSD2.0 Isothermal Master Mix

LAMP reactions made with the GspSSD2.0 Master Mix were of 25 μl volume made of DEPC water (5.5 μl), GspSSD2.0 Master Mix (15 μl), Primer Mix 10x (2.5 μl) Plasmid or DNA (2 μl). LAMP reactions were at 64° C for one hour in a GeneMate Mini Dry Bath with heated lid (BioExpress), followed by five minutes at 80° C to denature the polymerase complex and to stop the reaction. Then the tubes were hold in ice (circa 4°C) for a few minutes.

### Analytical sensitivity and specificity of LAMP assay

To determine the analytical sensitivity of the SFGR-LAMP assay, a recombinant plasmid carrying the 17kDa protein gene targeted sequence of *Rickettsia rickettsii* between the outer F3 and B3 LAMP primers was constructed with the QIAGEN Plasmid Mini Kit (Qiagen) following manufacturer’s protocol. To assess the limit of detection and reproducibility of the SFGR-LAMP assay, the obtained recombinant plasmid was serially diluted 10-fold and quantified with a NanoDrop UV-Vis Spectrophotometer (Thermo Scientific). Serial dilution concentrations were from 10ng to 1 fg. Furthermore, DNA from the crude preparations from a single rickettsia-infected tick were quantified and serially diluted to determine the limit of detection using the LAMP assay.

The inclusive specificity test included eight closely related *Rickettsia* spp. DNA from cultured strains of 6 SFG rickettsia species (*R*. *rickettsii*, *R*. *parkeri*, *R*. *africae*, *R*. *amblyommatis*, *R*. *conorii*, *R*. *montanensis*), a TRG rickettsia species (*R*. *felis*), and a TG rickettsia species (*R*. *typhi*) (Gillespie et al. 2009). DNA amplification from the specificity *Rickettsia* spp. assays were excised from gel and purified with a PureLink™ Quick Gel Extraction Kit (Invitrogen) and sequenced to verify the identity of the amplified LAMP target. All resulting sequences were queried using BLAST. An exclusive specificity panel was run using three *Rickettsia* spp. (*R*. *rickettsii*, *R*. *parkeri*, and *R*. *typhi*), three closely related bacteria species (*E*. *chaffeensis*, *Anaplasma phagocytophilum*, and *Coxiella burnetii*), and more distantly related bacteria species (*Escherichia coli*, and *Salmonella typhi*).

### Detection of SFG rickettsia in field-collected ticks and fleas

The SFGR-LAMP assay was further compared to end-point PCR by screening pools of field-collected unfed ticks from Oklahoma City and fleas collected from client-owned dogs and cats in two urban areas of Oklahoma. The collection protocol for fleas from client-owned dogs and cats was approved by the Institutional Animal Care and Use Committee (AG-16-16) at Oklahoma State University. The DNA extraction method used for the pools of field-collected ticks and fleas was previously described [21,22]. The analysis process for SFGR-LAMP assays on field-collected tick and flea samples was blinded. The results were independently assessed by two readers. An additional reader was not utilized as differences were not encountered between the two readers.

## Results

### Sensitivity and specificity of LAMP

**Fig 2:**
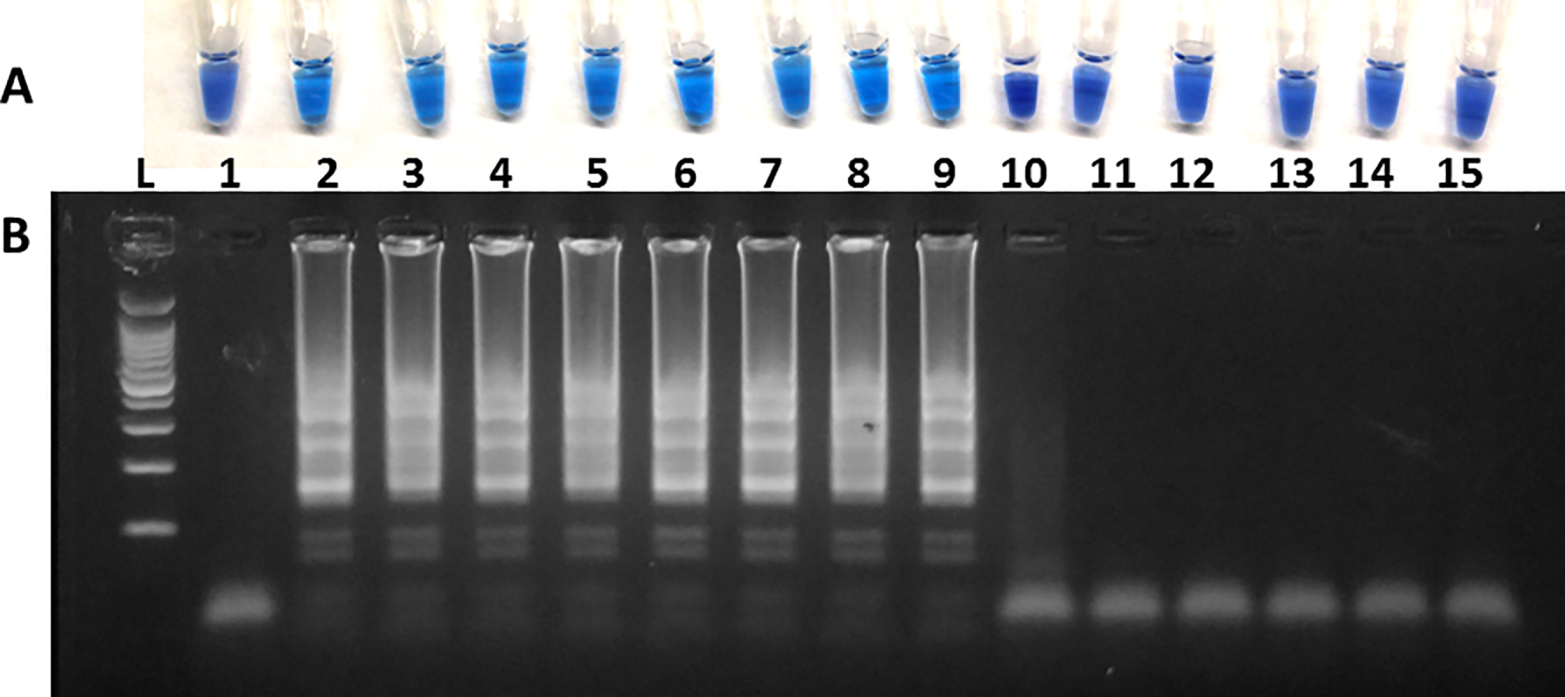
Specificity of SFGR-LAMP assay using Bst 2.0 WarmStart DNA polymerase. (A). Colorimetric visual detection of hydroxynaphol blue-based SFGR-LAMP reaction products inspected by the naked eye. The color changes from light blue in the positive reactions to dark blue to purple in the negative reactions. (B). Agarose gel electrophoresis. Lane 1: 100Kbp molecular ladder; 2: water control; 3: positive control (*R*. *rickettsii*); 4: *R*. *africae*; 5: *R*. *amblyommatis*; 6: *R*. *conorii*; 7: *R*. *felis*; 8: *R*. *montanensis*; 9: *R*. *parkeri*; 10: *R*. *rickettsii*; 11: *R*. *typhi*; 12: *E*. *chaffeensis*; 13: *A*. *phagocytophilum*; 14: *C*. *burnetii*; 15: *E*. *coli*; 16: *S*. *typhi*.

Successful LAMP amplification was achieved with either of the LAMP chemistries used, *Bst* 2.0 WarmStart DNA Polymerase (New England Biolabs, USA) and the GspSSD2.0 Isothermal Master Mix (Optigene, UK) (Figs 1 and 2) using primers listed in Table 1 and genomic DNA from a variety of SFG rickettsia species as well as crude DNA preparations of pooled field-collected ticks and pooled fleas from domestic dogs and cats. The sensitivity of the SFGR-LAMP assay was assessed using a reference plasmid as well as crude preparations from a single infected tick. The LAMP assay detected rickettsial DNA from the plasmid preparation down to the concentration of 0.00001 ng/μl (Fig 1). Using crude preparations of a single tick DNA, the SFGR-LAMP assay detected rickettsial DNA to a concentration of 0.01ng compared to 0.1ng detected with end-point PCR.

The analytical specificity of the LAMP assay was tested using genomic DNA from seven SFG rickettsia species, one Typhus group rickettsia, and several closely and more distantly related bacterial species (Fig 2). All six SFG rickettsia species (*R*. *rickettsii*, *R*. *parkeri*, *R*. *africae*, *R*. *amblyommatis*, *R*. *conorii*, *R*. *montanensis*) and *R*. *felis* (TRG rickettsia) were positive while *R*. *typhi* (TG rickettsia), *A*. *marginale* (not pictured), *A*. *phagocytophilum*, *E*. *chaffeensis*, *C*. *burnetii*, *E*. *coli*, *S*. *typhi*, and *S*. *enterococcus* (not pictured) were not detected (Fig 2).

### Detection of SFG rickettsia in field-collected ticks and fleas

Screening field samples with SFGR-LAMP assay was further compared to end-point PCR by screening 40 pools of field-collected ticks (n = 182 –pools consisted of 74 adult and 103 nymphal *A*. *americanum* and 5 adult *D*. *variabilis*) from Oklahoma City and 52 pools of fleas (total n = 153) collected from client-owned dogs and cats in two urban areas of Oklahoma (Fig 1, Table 2). PCR and LAMP detected rickettsia DNA in 72.5% (29/40 pools) of the same pools of field-collected ticks. Of the flea pools, 21 were positive by endpoint PCR and LAMP assay, respectively. The SFGR-LAMP assay detected rickettsia DNA in two pools which previously tested negative by PCR, one of which consisted of one *Pulex irritans* flea taken from a stray dog. Additionally, one pool (containing ‘*Candidatus* R. senegalensis’) was positive by end-point PCR but not the SFGR-LAMP assay.

**Table 2:** Comparison of end-point PCR and SFGR-LAMP assays using crude DNA extracted from pools of field-collected ticks and pools of fleas collected from client-owned dogs and cats in Oklahoma.

Samples	Species	Pools tested (total no.)	PCR positive (%)	LAMP positive (%)
Ticks	*A*. *americanum*	31 (177)	23 (74.2)	23 (74.2)
	*D*. *variabilis*	8 (8)	5 (62.5)	5 (62.5)
	*A*. *maculatum*	1 (1)	1 (100)	1 (100)
	**Total**	40	29 (72.5)	29 (72.5)
Fleas	*C*. *felis*	50 (151)	22 (44)	22 (44)
	*P*. *irritans*	2 (2)	0	1 (50)
	**Total**	52	22 (42.3)	23 (44.2)

## Discussion

Since its introduction in 2000, many studies have used some form of LAMP technology to detect vector-borne pathogens. The majority of the studies, however, developed LAMP assays to directly detect pathogen DNA in blood, sputum, feces, and skin of animals, including humans. By the end of 2016, only 2 LAMP studies detecting tick-transmitted bacterial pathogens had tested field-collected ticks during the development of their LAMP assays ((*Borrelia burgdorferi* [13] and *Ehrlichia ruminantium* [14]). No LAMP studies to date have screened fleas for rickettsial pathogens. This study introduces a functional SFGR-LAMP assay applicable as a surveillance tool to prompt initial screening of SFG and TRG rickettsia DNA in field-collected ticks and fleas.

Pools of field-collected ticks from an urban area of Oklahoma City, Oklahoma [22] and fleas from client-owned dogs [21] were screened by the SFGR-LAMP assay in this study, and results were compared to the published end-point PCR results. The SFG rickettsia species detected in field-collected ticks and fleas were *R*. *amblyommatis* (previously *R*. *amblyommii* [15], *R*. *montanensis*, and ‘*Candidatus* R. andeanae’ [22] and a TRG rickettsia, *R*. *felis* [21]. While both assays identified the same infected tick pools, LAMP detected two additional positive flea pools compared with end-point PCR. The pool of SFG-rickettsia infected *Ctenocephalides felis* fleas that was not detected by the SFGR-LAMP was most closely related to ‘*Candidatus* R. senegalensis’ [21]. Due to the recent characterization of this *Rickettsia* species, it is unclear whether ‘*C*.R. senegalensis’ is detected by 17kDa protein gene molecular assays. The screening capacity of the SFGR-LAMP assay indicates the capability of this assay to be implemented in resource-challenged countries for the initial screening of tick and/or flea samples. While a positive LAMP result indicates that (a) rickettsia species is/are present, further characterization using other molecular assays would have to follow for species determination [9]. The importance of the detection of a particular rickettsia species (and the scale of the public health response) is only apparent when the specific species is/are identified. While the pathogenicity of rickettsia species such as *R*. *prowazekii* and *R*. *rickettsii* are well-characterized, new rickettsial species with varying degrees of pathogenicity for mammalian hosts are increasingly being described. Future LAMP assays need to be developed that not only identify specific rickettsia species, but also separate pathogenic species from those with unknown pathogenicity to better inform regarding the threat [24].

To be useful in resource-challenged countries, field detection assays should be affordable, sensitive, specific, simple to use in a few steps and with minimal training, repeatable and rapid with results in less than 30 min, not dependent on a thermocycler, and deliverable to the end user as per World Health Organization guidelines [25]. Most of these components are present in LAMP assays [12] and were demonstrated during the development of this SFGR-LAMP assay in particular. LAMP technology is generally cheaper per sample than most of the currently used technologies for detection of pathogens in field-collected arthropods, i.e. qPCR and others [5, 9] because of less need for expensive equipment and complex expensive reagents. The developed SFGR-LAMP assay was comparatively sensitive to end-point PCR as it detected rickettsial DNA in several flea samples that tested negative using the end-point assay. Sensitivity of the SFGR-LAMP assay with crude DNA was considerably less sensitive than plasmid DNA–possibly due to the presence of inhibitors. The above result is in line with other LAMP studies which also demonstrated higher sensitivity when comparing end-point PCR [26–28]. Regarding specificity, the SFGR-LAMP assay detected six SFG and one TRG rickettsia species but did not detect other closely-related bacteria, including *R*. *typhi* (a TG rickettsia species) The ease of the assay was demonstrated by linking a one-step DNA extraction method with LAMP so that it only took 90 minutes from sample processing to record a visual result. The use of HNB as a colorimetric component as well as the relatively low incubation temperature allows this assay to be less equipment stringent than other molecular detection methods. The use of HNB as visual detection can be challenging to multiple readers, especially those who cannot distinguish purple from blue color. Additional efforts may be necessary to develop a consistent colorimetric assay using novel pH sensitive dyes [28,29] or even by adapting the reaction to be resolved with a lateral flow device. At the moment, the reaction can also be detected using qPCR (data not published). Altogether, this assay has potential to be transferable to be a field-based surveillance tool for tick and flea-borne rickettsial pathogens.

The surveillance of vector-borne pathogens in low-resource countries continues to rely on specialized molecular assays, especially end-point PCR or qPCR assays [5,9], which are costly and rely on the acquisition of equipment and resources, also funding from external partners even for basic surveillance. It remains difficult for developing nations seeking to conduct their own disease surveillance programs with a limited budget [30]. Several commercial LAMP assays (malaria (RealAMP [31]) and Human African Typanosoma (Loopamp *Trypanosoma brucei* Detection Kit [32])) have been developed and field-tested with several others in development. While effective for detecting pathogens present in the blood of animals or patients, several pathogens, including rickettsias, are difficult to detect in humans and animals and need alternative tools for conducting surveillance within a given region [5,9]. LAMP assays are an alternative way for screening vector-borne pathogens within a given local region and have the capacity to be implemented to assist with large, field-based vector-borne disease surveillance.

Since 2000, relatively few studies have used LAMP technology to test different species of field-collected arthropod samples for vector-borne pathogens. Due to rising costs and technical difficulties for screening local arthropod populations for particular vector-borne pathogens, the use of LAMP for arthropods to xenomonitor (presence/absence) in a given region is becoming more of a realization [33]. Based on all the LAMP characteristics, this assay has recently been used for routine screening of arboviruses (Western equine encephalitis virus, St. Louis encephalitis virus, and West Nile virus) in California [34], *Leishmania* sp. in sandflies in Ecuador [28] and tsetse flies for Human African Trypanosomiasis in various areas in central and eastern Africa [33,35]. There is a need for further development of low cost screening tools for tick and flea-borne pathogens for implementation in surveillance programs. While specific LAMP assays exist for tick and flea-borne bacterial (*Anaplasm*a, *Bartonella*, *Coxiell*a, *Borrelia*, *and Rickettsia*) and protozoal (*Babesia*, *Theileria*) species [13, 26,27,36–39], no studies have incorporated LAMP technologies into general surveillance strategies. Development of user-friendly rapid assays targeting pathogens transmitted by arthropods is easier and more cost effective to determine disease distribution within a given local area rather than testing infected people and/or their animals.

## References

[1] Acestor N, Cooksey R, Newton PN, Ménard D, Guerin PJ, Nakagawa J, et al Mapping the aetiology of non-malarial febrile illness in Southeast Asia through a systematic review—terra incognita impairing treatment policies. PLoS One. 2012; 7(9):e44269 doi: 10.1371/journal.pone.0044269 22970193 10.1371/journal.pone.0044269 PMC3435412

[2] Parola P, Paddock CD, Socolovschi C, Labruna MB, Mediannikov O, Kernif T, et al Update on tick-borne rickettsioses around the world: a geographic approach. Clin Microbiol Rev. 2013; 26(4):657–702. doi: 10.1128/CMR.00032-13 24092850 10.1128/CMR.00032-13 PMC3811236

[3] Chiteka I, Dumler JS. Neglected bacterial zoonoses. Clin Microbiol Infect. 2015; 21(5):404–415. doi: 10.1016/j.cmi.2015.04.022 25964152 10.1016/j.cmi.2015.04.022 PMC4466158

[4] Brown LD, Macaluso KR. *Rickettsia felis*, an emerging flea-borne rickettsiosis. Curr Trop Med Rep. 2016; 3:27–39. doi: 10.1007/s40475-016-0070-6 27340613 10.1007/s40475-016-0070-6 PMC4870301

[5] Richards AL. Worldwide detection and identification of new and old rickettsiae and rickettsial diseases. FEMS Immunol Med Microbiol. 2012; 64(1):107–110. doi: 10.1111/j.1574-695X.2011.00875.x 22067055 10.1111/j.1574-695X.2011.00875.x

[6] Paris DH, Dumler JS. State of the art of diagnosis of rickettsial diseases: the use of blood specimens for diagnosis of scrub typhus, spotted fever group rickettsiosis, and murine typhus. Curr Opin Infect Dis. 2016; 29(5):433–439. doi: 10.1097/QCO.0000000000000298 27429138 10.1097/QCO.0000000000000298 PMC5029442

[7] Prabhu M, Nicholson WL, Roche AJ, Kersh GJ, Fitzpatrick KA, Oliver LD, et al Q fever, spotted fever group, and typhus group rickettsioses among hospitalized febrile patients in northern Tanzania. Clin Infect Dis. 2011; 53(4):e8–15. doi: 10.1093/cid/cir411 21810740 10.1093/cid/cir411 PMC3148261

[8] Noden BH, Tshavuka FI, van der Colf BE, Chipare I, Wilkinson R. Exposure and risk factors to *Coxiella burnetii*, spotted fever group and typhus group Rickettsiae, and *Bartonella henselae* among volunteer blood donors in Namibia. PLoS One. 2014; 9(9):e108674 doi: 10.1371/journal.pone.0108674 25259959 10.1371/journal.pone.0108674 PMC4178180

[9] Luce-Fedrow A, Mullins K, Kostik AP, St John HK, Jiang J, Richards AL. Strategies for detecting rickettsiae and diagnosing rickettsial diseases. Future Microbiol. 2015; 10(4):537–564. doi: 10.2217/fmb.14.141 25865193 10.2217/fmb.14.141

[10] Notomi T, Okayama H, Masubuchi H, Yonekawa T, Watanabe K, Amino N, et al Loop-mediated isothermal amplification of DNA. Nucleic Acids Res. 2000; 28(12):E63 10871386 10.1093/nar/28.12.e63 PMC102748

[11] Mori Y, Kanda H, Notomi T. Loop-mediated isothermal amplification (LAMP): recent progress in research and development. J Infect Chemother. 2013; 19(3):404–411. doi: 10.1007/s10156-013-0590-0 23539453 10.1007/s10156-013-0590-0 PMC7088141

[12] Njiru ZK. Loop-mediated isothermal amplification technology: towards point of care diagnostics. PLoS Negl Trop Dis. 2012; 6(6):e1572 doi: 10.1371/journal.pntd.0001572 22745836 10.1371/journal.pntd.0001572 PMC3383729

[13] Yang J, Guan G, Niu Q, Liu Z, Li Y, Liu J, et al Development and application of a loop-mediated isothermal amplification assay for rapid detection of *Borrelia burgdorferi s*. *l*. in ticks. Transbound Emerg Dis. 2013; 60(3):238–244. doi: 10.1111/j.1865-1682.2012.01335.x 22587441 10.1111/j.1865-1682.2012.01335.x

[14] Nakao R, Stromdahl EY, Magona JW, Faburay B, Namangala B, Malele I et al Development of loop-mediated isothermal amplification (LAMP) assays for rapid detection of *Ehrlichia ruminantium*. BMC Microbiol. 2010; 10:296 doi: 10.1186/1471-2180-10-296 21087521 10.1186/1471-2180-10-296 PMC3000401

[15] Karpathy SE, Slater KS, Goldsmith CS, Nicholson WL, Paddock CD. *Rickettsia amblyommatis* sp. nov., a spotted fever group *Rickettsia* associated with multiple species of *Amblyomma* ticks in North, Central and South America. Int J Syst Evol Microbiol. 2016; 66(12):5236–5243. doi: 10.1099/ijsem.0.001502 27638476 10.1099/ijsem.0.001502 PMC5893998

[16] Gillespie JJ, Ammerman NC, Beier-Sexton M, Sobral BS, Azad AF. Louse- and flea-borne rickettsioses: biological and genomic analyses. Vet Res. 2009; 40(2):12 doi: 10.1051/vetres:2008050 19036234 10.1051/vetres:2008050 PMC2695025

[17] Salazar, J.L. Detection of tick-borne pathogens in lab reared tick colonies and wild populations. Master’s thesis. 2015. Oklahoma State University.

[18] Martin J. Distribution of ticks of medical and veterinary importance along the Chisholm Trail and development of a molecular assay to detect Rickettsia spp. in field-collected ticks in Oklahoma. Master’s thesis. 2016. Oklahoma State University, Stillwater, OK.

[19] Tzianabos T, Anderson BE, McDade JE. Detection of *Rickettsia rickettsii* DNA in clinical specimens by using polymerase chain reaction technology. J. Clin. Microbiol. 1989; 27: 2866–2868. 2512328 10.1128/jcm.27.12.2866-2868.1989 PMC267147

[20] Jiang J, Stromdahl EY, Richards AL. Detection of *Rickettsia parkeri* and *Candidatus* Rickettsia andeanae in *Amblyomma maculatum* Gulf Coast ticks collected from humans in the United States. Vector Borne Zoonotic Dis. 2012; 12(3):175–182. doi: 10.1089/vbz.2011.0614 22022815 10.1089/vbz.2011.0614

[21] Noden BH, Davidson S, Smith JL, Williams F. First detection of *Rickettsia typhi* and *R*. *felis* in fleas collected from client-owned companion animals in the southern Great Plains. J Med Entomol. 2017a; 54(4):1093–1097. doi: 10.1093/jme/tjx069 28399210 10.1093/jme/tjx069

[22] Noden BH, Loss SR, Maichak C, Williams F. Risk of encountering ticks and tick-borne pathogens in a rapidly growing metropolitan area in the U.S. Great Plains. Ticks Tick Borne Dis. 2017b; 8(1):119–124. doi: 10.1016/j.ttbdis.2016.10.007 27773826 10.1016/j.ttbdis.2016.10.007

[23] Labruna MB, Whitworth T, Horta MC, Bouyer DH, McBride JW, Pinter A, et al *Rickettsia* species infecting *Amblyomma cooperi* ticks from an area in the state of São Paulo, Brazil, where Brazilian spotted fever is endemic. J Clin Microbiol. 2004; 42(1):90–98. doi: 10.1128/JCM.42.1.90-98.2004 14715737 10.1128/JCM.42.1.90-98.2004 PMC321730

[24] Beier-Sexton M, Driscoll TP, Azad AF, Gillespie JJ. The Family Rickettsiaceae In: Goldman E, Green LH, editors. Practical Handbook of Microbiology, 3^rd^ edition. Boca Raton, Florida: CRC Press; 2015 pp. 547–566.

[25] Mabey D, Peeling RW, Ustianowski A, Perkins MD. Diagnostics for the developing world. Nat Rev Microbiol 2004; 2:231–240. doi: 10.1038/nrmicro841 15083158 10.1038/nrmicro841

[26] Pan L, Zhang L, Wang G, Liu Q. Rapid, simple, and sensitive detection of the ompB gene of spotted fever group rickettsiae by loop-mediated isothermal amplification. BMC Infect Dis. 2012; 12:254 doi: 10.1186/1471-2334-12-254 23057497 10.1186/1471-2334-12-254 PMC3524767

[27] Pan L, Zhang L, Fan D, Zhang X, Liu H, Lu Q et al Rapid, simple and sensitive detection of Q fever by loop-mediated isothermal amplification of the htpAB gene. PLoS Negl Trop Dis. 2013; 7(5):e2231 doi: 10.1371/journal.pntd.0002231 23696915 10.1371/journal.pntd.0002231 PMC3656153

[28] Nzelu CO, Gomez EA, Cáceres AG, Sakurai T, Martini-Robles L, Uezato H, et al Development of a loop-mediated isothermal amplification method for rapid mass-screening of sand flies for *Leishmania* infection. Acta Trop. 2014; 132:1–6. doi: 10.1016/j.actatropica.2013.12.016 24388795 10.1016/j.actatropica.2013.12.016

[29] Lucchi NW, Ljolje D, Silva-Flannery L, Udhayakumar V. Use of malachite green-loop mediated isothermal amplification for detection of *Plasmodium* spp. parasites. PLoS One. 2016; 11(3):e0151437 doi: 10.1371/journal.pone.0151437 26967908 10.1371/journal.pone.0151437 PMC4788150

[30] Sokhna C, Mediannikov O, Fenollar F, Bassene H, Diatta G, Tall A, et al Point-of-care laboratory of pathogen diagnosis in rural Senegal. PLoS Negl Trop Dis. 2013; 7(1):e1999 doi: 10.1371/journal.pntd.0001999 23350001 10.1371/journal.pntd.0001999 PMC3547848

[31] Patel JC, Lucchi NW, Srivastava P, Lin JT, Sug-Aram R, Aruncharus S et al Field evaluation of a real-time fluorescence loop-mediated isothermal amplification assay, RealAmp, for the diagnosis of malaria in Thailand and India. J Infect Dis. 2014; 210(8):1180–1187. doi: 10.1093/infdis/jiu252 24795480 10.1093/infdis/jiu252 PMC6373533

[32] Mitashi P1, Hasker E, Ngoyi DM, Pyana PP, Lejon V, Van der Veken W et al Diagnostic accuracy of loopamp *Trypanosoma brucei* detection kit for diagnosis of human African trypanosomiasis in clinical samples. PLoS Negl Trop Dis. 2013; 7(10):e2504 doi: 10.1371/journal.pntd.0002504 24147176 10.1371/journal.pntd.0002504 PMC3798548

[33] Cunningham LJ, Lingley JK, Haines LR, Ndung'u JM, Torr SJ, Adams ER. Illuminating the prevalence of *Trypanosoma brucei* s.l. in *Glossina* using LAMP as a tool for xenomonitoring. PLoS Negl Trop Dis. 2016; 10(2):e0004441 doi: 10.1371/journal.pntd.0004441 26890882 10.1371/journal.pntd.0004441 PMC4758712

[34] Wheeler SS, Ball CS, Langevin SA, Fang Y, Coffey LL, Meagher RJ. Surveillance for Western Equine Encephalitis, St. Louis Encephalitis, and West Nile viruses using reverse transcription loop-mediated isothermal amplification. PLoS One. 2016; 11(1):e0147962 doi: 10.1371/journal.pone.0147962 26807734 10.1371/journal.pone.0147962 PMC4726549

[35] Laohasinnarong D, Goto Y, Asada M, Nakao R, Hayashida K, Kajino K, et al Studies of trypanosomiasis in the Luangwa valley, north-eastern Zambia. Parasit Vectors. 2015; 8:497 doi: 10.1186/s13071-015-1112-y 26419347 10.1186/s13071-015-1112-y PMC4589067

[36] Pan L, Zhang L, Wang G, Liu Q, Yu Y, Wang S et al Rapid, simple, and sensitive detection of *Anaplasma phagocytophilum* by loop-mediated isothermal amplification of the msp2 gene. J Clin Microbiol. 2011; 49(12):4117–4120. doi: 10.1128/JCM.01085-11 21976758 10.1128/JCM.01085-11 PMC3232955

[37] Dittrich S, Castonguay-Vanier J, Moore CE, Thongyoo N, Newton PN, Paris DH. Loop-mediated isothermal amplification for *Rickettsia typhi* (the causal agent of murine typhus): problems with diagnosis at the limit of detection. J Clin Microbiol. 2014; 52(3):832–838. doi: 10.1128/JCM.02786-13 24371248 10.1128/JCM.02786-13 PMC3957756

[38] Yang Y, Li Q, Wang S, Chen X, Du A. Rapid and sensitive detection of *Babesia bovis* and *Babesia bigemina* by loop-mediated isothermal amplification combined with a lateral flow dipstick. Vet Parasitol. 2016; 219:71–76. doi: 10.1016/j.vetpar.2016.02.004 26921043 10.1016/j.vetpar.2016.02.004

[39] Gomes J, Santos M, Amaro A, Pereira da Fonseca I, Santos-Gomes G, Inácio J. A field evaluation of an isothermal DNA amplification assay for the detection of *Theileria annulata* infection in cattle. Mol Cell Probes. 2017; 31:61–64. doi: 10.1016/j.mcp.2016.12.006 28013043 10.1016/j.mcp.2016.12.006

